# Glycated Hemoglobin is a Significant Predictor of Femoral, but Not of Carotid or Popliteal, Intima-Media Thickness in Adolescents with Type 1 Diabetes: A Case-Series Study

**DOI:** 10.1155/2023/6471597

**Published:** 2023-03-31

**Authors:** Hung-Chi Ho, Fu-Sung Lo, Jen-Kuang Lee, Wen-Yu Tsai, Ta-Chen Su

**Affiliations:** ^1^Department of Internal Medicine and Big Data Center, China Medical University Hospital and College of Medicine, China Medical University, Taichung 404327, Taiwan; ^2^Division of Pediatric Endocrinology, Department of Pediatrics, Chang Gung Memorial Hospital, Chung Gung University College of Medicine, Kweishan, Guishan District, Taoyuan City 33423, Taiwan; ^3^Division of Cardiology, Department of Internal Medicine, National Taiwan University Hospital, Zhongzheng District, Taipei 100225, Taiwan; ^4^Department of Pediatrics, National Taiwan University College of Medicine, Zhongzheng District, Taipei 10617, Taiwan; ^5^Department of Environmental and Occupational Medicine, National Taiwan University Hospital, Zhongzheng District, Taipei 100225, Taiwan

## Abstract

In healthy adults, the association between the glycated hemoglobin A1c (HbA1c) level and intima-media thickness (IMT) is stronger in the femoral artery than that in the carotid artery. However, whether this differential strength of association also applies to adolescents with type 1 diabetes (T1D) is unknown. Therefore, this study aimed to examine whether IMT increases in extracarotid arteries (specifically in the lower extremities) prior to the carotid artery. In total, 286 adolescents with T1D (15.9 ± 4.9 years; 42.0% male participants) were enrolled, and the B-mode ultrasonographic measurement of IMT in the carotid, femoral, and popliteal arteries was performed. Cardiovascular risk factors, including blood pressure (BP), body mass index, lipid levels, and glycemic parameters, were evaluated. To evaluate the site-dependent relationship between IMT and cardiovascular risk factors, a linear mixed-effects model was developed with repeated IMT measurements at various arterial sites as fixed effects and participants as random effects. Glycemic parameters, lipids, uric acid, high-sensitivity C-reactive protein, and advanced glycation end-products were some cardiovascular risk factors that worsened with increasing HbA1c levels. Patients with a higher HbA1c level (>10% vs. ≤10%) had thicker IMT in the femoral artery but not in the carotid or popliteal arteries. Patients with poorer diabetic control exhibited significant changes in certain cardiovascular functions, including central systolic BP, left ventricular (LV) ejection time, LV dp/dt max, stroke volume, and brachial artery compliance. A standard mediation analysis revealed that none of the aforementioned cardiovascular functions mediated the relationship between higher HbA1c level and greater femoral IMT. In adolescents with T1D, cardiovascular risk factors deteriorate with worsening blood glucose control. In the early stages of T1D, femoral IMT may serve as a more sensitive surrogate marker for hyperglycemia-induced subclinical atherosclerosis, an effect that may not be mediated by alterations in cardiovascular functions.

## 1. Introduction

A large cohort study (*n* = 45,595) revealed that men with type 1 diabetes (T1D) aged 45–55 years have an absolute cardiovascular risk comparable to that of men 10–15 years older in the general population, whereas women with T1D have an even higher risk [1]. Given that the hard-endpoint diseases may develop at an accelerated pace in T1D, it is reasonable to prioritize the detection of subclinical atherosclerosis in younger patients. As demonstrated by the previous case-control studies, these T1D patients also have a greater carotid intima-media thickness (IMT) [2–4]. As a surrogate marker for subclinical atherosclerosis, carotid IMT has been associated with cardiovascular risk factors and outcomes [5–7]. On high-resolution B-mode ultrasonography, IMT is defined as the distance between the blood-intima interface and the media-adventitia interface on the artery's distal wall. Owing to its noninvasive nature and technological simplicity, carotid ultrasonography is widely employed in large-scale population studies to estimate future cardiovascular risk or magnitude of treatment effects [8, 9]. Although the aforementioned evidence has been deemed credible for middle-aged and elderly patients, data for younger individuals are limited.

Given that hyperglycemia is a defining characteristic of T1D, the question naturally arises as to whether hyperglycemia influences IMT, independently of other conventional risk factors. According to the previous studies conducted primarily on adults with or without diabetes, only diabetes status (diabetic or not), but not the blood glucose level, was reported as an independent predictor of carotid IMT [10, 11]. The susceptibility of vascular endothelium to hyperglycemia-induced atherogenesis varies between arteries and between the segments within the same artery [12]. Therefore, we hypothesize that the IMT will increase in extracarotid arteries (specifically in the lower extremities) prior to that in the carotid artery. Therefore, to mitigate the potential nonhyperglycemia-related confounding effects of factors such as aging, this study aimed to test this hypothesis in a cohort of adolescents (mean age, 15.9 ± 4.9 years) with T1D who underwent vascular ultrasonography to measure the IMT in the carotid, femoral, and popliteal arteries.

## 2. Materials and Methods

### 2.1. Study Protocol and Population

This Subclinical Cardiovascular Disease in Type 1 Diabetes Mellitus (SCVD-T1DM) cohort study was conducted in Taiwan. We enrolled patients who fulfilled the American Diabetes Association's diagnostic criteria for T1D [13] and their age- and sex-matched nondiabetic siblings as controls. Individuals with cardiovascular disease at the baseline were excluded. The objective of the SCVD-T1DM study was to examine the predictors of atherosclerosis and to explore the association between subclinical atherosclerosis and diabetes-related comorbidities using a case-control design. The study was conducted in accordance with the Declaration of Helsinki, and its design was approved by the Institutional Review Board of National Taiwan University Hospital, Taiwan (IRB# 201205043RIB). No animals were used in this study. All participants provided informed consent prior to participation in the study.

From 2011 to 2013, we recruited 286 young patients with T1D and 106 age- and sex-matched nondiabetic sibling controls (aged 6–40 years) from three Taiwanese medical centers and the Taiwan Association for Diabetic Children. Each participant underwent thorough examination and filled out a questionnaire regarding workplace stress, self-esteem, sleep problems, and general psychosocial health. BP, height, weight, and waist circumference were measured in accordance with the standard protocols. After 10–12 h of overnight fasting, blood samples were collected from each participant. We measured serum albumin, fasting glucose, HbA1c, insulin, lipids, high-sensitivity C-reactive protein (hs-CRP), liver enzymes, and electrolyte levels and assessed kidney function and red blood cell count. Urine samples from the first morning void were collected and analyzed for protein, glucose, and electrolyte levels. Genomic DNA was extracted from the peripheral blood using standard procedures.

### 2.2. Noninvasive Cardiovascular Assessments: Measuring IMT in the Carotid and Lower Extremity Arteries

The IMT of the extracranial carotid artery was measured using high-resolution B-mode ultrasound (3.5–10 MHz; GE Vivid ultrasound system, Horten, Norway). The measurement protocol was previously published [14]. On ultrasonography, the IMT appears 20% thinner than that on histology when measured on the near wall [15]. Consequently, IMT measurements were acquired by tracing the blood-intima and media-adventitia interfaces on the far wall. A total length of 1 cm was measured using the leading edge-to-leading edge method in the presence of the double-line sign, ensuring that the vessel was imaged through its truest diameter. The mean and maximum IMT values were generated automatically by the software. The six carotid artery segments measured included the distal 1 cm of the common carotid artery (CCA), the carotid bulb (Bulb), and the proximal 1 cm of the internal carotid artery (ICA) bilaterally. All images and measurements were digitally archived for future review and analysis. The maximum IMT of the carotid artery was calculated as the mean of the six segments' maximum IMTs (bilateral CCAs, Bulbs, and ICAs). The mean IMT of the carotid artery was calculated by averaging the six respective segment means.

IMT values in the lower extremity were obtained for the distal 1 cm of the common femoral artery (CFA) and the popliteal artery (PLA). Two consecutive IMT measurements were taken 2 weeks apart on 30 randomly chosen participants, and the intraexaminer correlation coefficient of reliability (ICCR) was found to be excellent at 0.99 [16].

### 2.3. Assessment of Cardiovascular Functions: The DynaPulse System

Using an upper-arm cuff to apply pressure to the brachial artery, pulse waveform signals were obtained and recorded by an oscillometric device called the DynaPulse BP monitor (model 2000A, Pulse Metric Inc., Vista, CA) [17]. This device records and analyzes cuff pulsation signals during BP measurements using a pulse oscillometric technique. It employs a pattern recognition method to evaluate BP, eliminates the reliance on audible sounds that may lead to interexaminer inconsistency, and enables objective and automatic measurement of BP. Let *t*_pp_ and d*P* denote the time interval and amplitude between the peak positive and peak negative pressure derivatives, respectively; *D*_0_ is the diameter of the brachial artery, which can be estimated based on a person's sex, height, weight, and mean BP; and *L*_*c*_ is the width of the elective cuff. Assuming a physical model of a straight-tube brachial artery and the T-tube aortic system [18], the compliance and distensibility of the brachial artery can be calculated as a function of *t*_pp_, d*P*, *D*_0_, and *L*_*c*_. These parameters have been validated noninvasively [19] and invasively [20] and are associated with conventional cardiovascular risk factors in young healthy adults [21]. In addition, these parameters have been used in scientific research [16] to derive hemodynamic parameters, such as the cardiac output, stroke volume, and maximum rate of increase in left ventricular pressure (LV d*P*/d*t* max). The data were transmitted via the Internet to a remote analysis center.

### 2.4. Statistical Analysis

Continuous variables are presented as means (standard deviations), and binary variables are presented as percentages according to the three predetermined brackets of HbA1c values (≤7%; >7% and ≤10%; and >10%). Trend tests for continuous and binary variables in relation to the HbA1c levels were analyzed using linear and logistic regression models, respectively, while accounting for sex and age. The assumption of normality of the data was not required for these variables. To determine the site-specific atherogenic effects of hyperglycemia, mean and maximum IMT values were calculated for the carotid (CCA, Bulb, ICA), femoral (CFA), and popliteal (PLA) arteries.

A linear mixed-effects model with repeated measurements as fixed effects and participants as random effects were fitted to the data to account for between-participant heterogeneity in IMT values and within-participant correlation between the repeated IMT measurements [22]. The predictors of IMT were separately analyzed at various arterial sites (carotid, femoral, and popliteal). At each arterial site, repeated IMT measurements were simultaneously regressed on the predictors of interest as fixed effects. The repeated measurements as both fixed and random effects were fitted with a general unstructured covariance matrix. As the mean IMT may vary between the segments, a categorical “Segments” variable was introduced as both fixed and random effects for each arterial site. At the carotid site, the “Segments” variable consisted of six levels representing bilateral CCAs, Bulbs, and ICAs; at the femoral site, two levels representing bilateral CFAs; and at the popliteal site, two levels representing bilateral PLAs. At each segment, the mean and maximum IMT were analyzed separately as the response variable. A maximum likelihood estimation method was applied to estimate the fixed effects and covariance matrix and to infer their statistical significance. Model diagnostics were performed by checking the normality of the random effects, residual analysis, and influence analysis.

Path diagrams based on structural equation modeling [23, 24] were used to examine the mediation of cardiovascular measures, as assessed using the DynaPulse system, on the association between the HbA1c levels and femoral IMT. For a variable of interest to serve as a mediator, each of the following three path regression coefficients must first be statistically significant: HbA1c, as a predictor of femoral IMT; HbA1c, as a predictor of the mediator; and the mediator, as a predictor of femoral IMT. Then, the overall mediation analysis continues by regressing femoral IMT on HbA1c both with and without adjusting for the mediator. Depending on whether or not the mediator is adjusted for, the path regression coefficient for HbA1c is interpreted as either a direct or a total effect, respectively. An indirect effect is obtained by subtracting the direct effect from the total effect. Mediation is considered to exist if the indirect effect is significant. For each of the DynaPulse measures that served as potential mediators, we calculated the three path regression coefficients, and if all were significant, the total, direct, and indirect effects were calculated. To avoid making distributional assumptions, we drew 2000 bootstrap samples to generate 95% confidence intervals.

All statistical analyses were performed with RStudio version 2022.12.0 and R version 4.2.2 R Foundation for Statistical Computing, Vienna, Austria. The linear mixed-effects model was fitted using the “lme()” function in the nlme package. The mediation analysis was conducted using the lavaan package.

## 3. Results

### 3.1. Baseline Characteristics

Table 1 outlines the baseline characteristics and biochemical findings of the entire study population grouped according to the predetermined HbA1c levels (≤7%; >7% and ≤10%; and >10%). Patients with T1D had a mean age of 15.9 ± 4.9 years (range, 5.5–32.6 years) and an average disease onset and duration of 8.5 ± 4.1 years and 7.5 ± 4.7 years, respectively. Moreover, male individuals accounted for 42.0% of the cohort. The mean HbA1c level was 8.4% ± 1.9% (67.8 ± 20.2 mmol/mol). Anthropometric measurements and BPs were within the normal range: body mass index (BMI), 20.2 ± 3.3 kg/m^2^; waist, 69.5 ± 9.4 cm; systolic BP, 115 ± 11 mmHg; and diastolic BP, 68 ± 7 mmHg. Variables that changed with the HbA1c level (*P* < 0.05) included the duration of T1D, fasting glucose, total cholesterol (TC), non-high-density lipoprotein (HDL) cholesterol (non-HDL-C), low-density lipoprotein (LDL) cholesterol (LDL-C), small dense LDL-C (sd-LDL-C), triglycerides, uric acid (decreasing), hs-CRP, and advanced glycation end-product (AGE).

### 3.2. IMT in the Carotid and Lower Extremity Arteries

Table 2 lists the mean and maximum IMT by the HbA1c levels and segments of the carotid and lower extremity arteries. The maximum IMT of the left ICA, left Bulb, right CFA, and right PLA increased with increasing HbA1c (*P* < 0.05). Similar associations were observed for the mean IMT of the left Bulb, right CFA, bilateral CFA, right PLA, and bilateral PLA. The prevalence of the triple line pattern increased with HbA1c in the PLA. In brief, positive associations between the IMT and the HbA1c levels were more prominent in the lower extremity arteries than in the carotid arteries.

**Figure 1:**
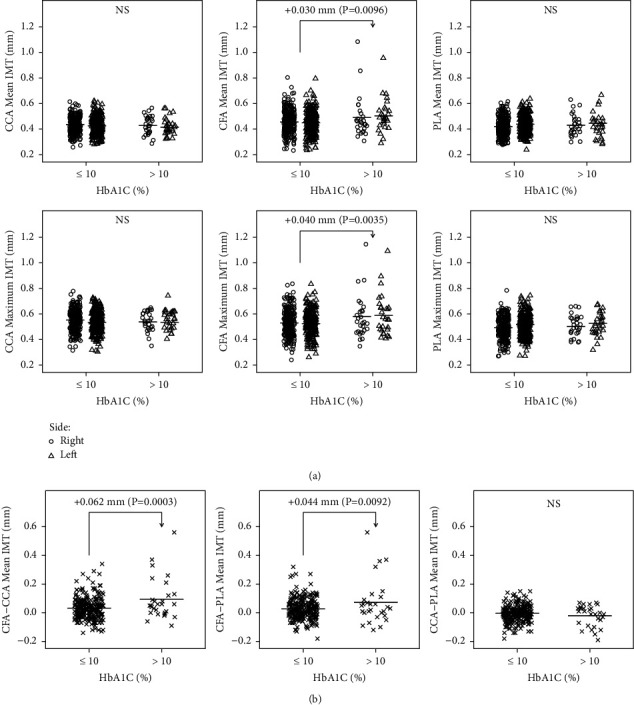
(a) HbA1c category (≤10% versus >10%) and bilateral IMTs (mean and maximum) at various arterial sites in adolescents with T1D. The sample means are indicated by the horizontal bars. (b) Paired difference values are on the vertical axis. CFA-CCA mean IMT represents the difference between the CFA and CCA mean IMT values. CCA, common carotid artery; CFA, common femoral artery; IMT, intima-media thickness; NS, not significant; PLA, popliteal artery.

Univariate analysis revealed a highly significant association between the HbA1c category (≤10% versus >10%) and IMT (mean and maximum) of the bilateral CFAs (Figure 1). It was estimated that the higher HbA1c (>10% versus ≤10%) levels were associated with a 0.030-mm increase in the mean IMT (95% CI, 0.007–0.054 mm; *P*=0.0096) and a 0.040-mm increase in the maximum IMT (95% CI, 0.013–0.067 mm; *P*=0.0035) of the bilateral CFAs. No significant associations were observed for the CCAs and PLAs.

Table 3 shows the estimated fixed-effect coefficients for the predictors of IMT at various arterial sites based on a linear mixed-effects model. Segment and sex had significant effects at the carotid site, with male individuals having greater IMT (mean IMT: 1.98 × 10^−2^ mm, *P* < 0.001; maximum IMT: 2.25 × 10^−2^ mm, *P* < 0.001). Age had a universally significant effect across all arterial sites, with older age being associated with a thicker IMT. In contrast, waist had a variable impact on IMT. Higher HbA1c (>10% vs. ≤10%) levels were associated with a greater IMT only in the CFA but not at the other sites. In the CFA, higher HbA1c levels were associated with a 3.95 × 10^−2^ mm (95% CI, 0.88 × 10^−2^–7.02 × 10^−2^ mm; *P* = 0.0106) and a 4.89 × 10^−2^ mm (95% CI, 1.34 × 10^−2^–8.45 × 10^−2^ mm; *P* = 0.0063) increase in the mean and maximum IMT, respectively.

### 3.3. Cardiac and Vascular Parameters Measured with DynaPulse

Cardiovascular functions were measured using the DynaPulse system (Table 4). Variables that increased alongside HbA1c included systolic BP, diastolic BP, central systolic BP, mean artery pressure, heart rate, LV ejection time, LV dp/dt max, stroke volume, and brachial artery compliance.

### 3.4. Mediation Analysis

Initial path regression analyses revealed the following significant associations: HbA1c as a predictor of femoral IMT; and HbA1c as a predictor of certain DynaPulse measures which in turn serve as a potential mediator, including central systolic BP, LV ejection time, LV dp/dt max, stroke volume, and brachial artery compliance. However, none of the potential DynaPulse mediators were significantly associated with femoral IMT, which is in line with the negligible indirect effects obtained from the overall mediation analysis (the results are not presented to save space).

## 4. Discussion

The present study demonstrated that adolescents with T1D had a greater IMT with increasing HbA1c in the CFA but not at the carotid or popliteal sites. Sex (the number of male individuals was more than that of female individuals), age, HDL-C (inverse association; CCA only), and urine albumin/creatinine ratio (CCA only) were independent predictors of IMT in the CCA, Bulb, and ICA, whereas age and HbA1c (CFA only) predicted IMT in the CFA and PLA.

These population-level associations were derived from a young cohort of adolescents with T1D; therefore, the analyses were unaffected by the confounding effects of multiple comorbidities typically observed in older populations. These findings demonstrate the site-specific effects of hyperglycemia on the early stages of atherosclerosis, with the effects being more pronounced in the femoral artery than in the carotid or popliteal arteries, as measured by IMT in vascular ultrasonography.

The simultaneous ultrasonographic assessment of IMT at multiple arterial sites extends previous autopsy findings obtained from young adults (mean age, 32 years) who died primarily from external causes, such as accidents or homicide [25]. This indicates a consistent association between age and subclinical atherosclerosis among adolescents (mean age, 16 years). In the past two decades, accumulating evidence has suggested a robust association between age and coronary artery calcification (a surrogate marker for coronary atherosclerosis) in apparently healthy individuals of a wide age range [26]. Collectively, these findings support and highlight the detrimental effects of aging on the underlying systemic atherosclerosis.

In this study, age and the blood glucose level were independently associated with IMT in adolescents with T1D. These findings are consistent with those of previous research conducted primarily on adult populations [27–29]. Blood glucose was identified as a strong independent predictor of IMT in the femoral artery in addition to conventional and modifiable risk factors. In healthy individuals, femoral IMT was reported to be comparable with that of the carotid and popliteal sites [30]. Therefore, the significant increase in the femoral IMT in adolescents with T1D (as demonstrated herein) suggests that femoral atherosclerosis develops earlier here than that in the carotid and popliteal arteries. This result is consistent with the findings of a previous study on healthy adult populations [31]. The site-dependent strength of association between IMT and blood glucose suggests that different segments of the arterial tree are susceptible to hyperglycemia-induced atherosclerosis to different degrees. The observed thickening of the femoral IMT in adolescents with T1D and its strong association with blood glucose, as measured by the HbA1c level, suggests that this site is more sensitive than carotid or popliteal sites to hyperglycemia and, therefore, more appropriate for early detection of atherosclerosis.

Atherosclerosis tends to develop at arterial sites with low shear stress and high flow oscillation from a hemodynamic standpoint. In a previous study, modern high-resolution magnetic resonance imaging revealed that the femoral artery possesses these characteristics to a greater degree than the carotid and brachial arteries [32]. This may be attributed to the fact that the carotid artery supplies an organ that requires a relatively constant blood supply, whereas the femoral artery must accommodate a four-to-eight fold increase in the blood flow during leg exercise. In addition, the subendothelial accumulation of LDL particles (which can be modified by hyperglycemia-induced oxidation and glycation) is a significant contributor to endothelial dysfunction and a characteristic of atherosclerosis [33]. These findings suggest that a combination of low shear stress, high oscillatory flow, and hyperglycemia-induced modification of LDL particles may accelerate IMT thickening in the femoral artery of patients with T1D. Consequently, measuring the IMT in the femoral and carotid arteries can aid in earlier detection of atherosclerosis's initial changes.

We found no significant independent associations between the carotid IMT and BP or lipid levels using multiple regression analysis with adjustment for baseline covariates. This contradicts the results of the previous cross-sectional studies [34–36], which reported independent associations between carotid IMT and the two risk factors in patients of the same age groups (mean age, 11–14 years). This discrepancy may be attributed to the fact that BP, lipid levels, and carotid IMT increase with age in healthy individuals before adulthood [30]. As observed in a previous study, a multiple regression analysis exploring the impact of BP or lipids on carotid IMT without simultaneously adjusting for age as a confounding factor would result in the incorrect identification of these risk factors as independent predictors [35]. Another possible explanation is that vascular changes associated with aging may not be detectable in young patients like those enrolled in the present study.

Patients with higher HbA1c levels also showed significant changes in certain cardiovascular functions, as assessed with the DynaPulse system, including systolic BP, diastolic BP, central systolic BP, mean arterial pressure, heart rate, LV ejection time, LV d*p*/d*t* max, stroke volume, and brachial artery compliance. Our mediation analysis revealed that the effect of HbA1c on IMT was not mediated by changes in cardiovascular functions. In the context of T1D, researchers have investigated the potential determinants of arterial stiffness, which is another cardiovascular function, as measured by pulse wave velocity (PWV). In a case-control study (199 T1D cases vs. 178 controls), the presence of T1D had a significant association with a higher carotid-radial PWV, but a trend towards significance was only noted for carotid-femoral PWV [37]. In a large cohort of 1809 youth patients with T1D, higher HbA1c levels were associated with carotid-femoral PWV [38]. As our analysis did not support the hypothesis that any of the cardiovascular functions serves as a mediator of HbA1c as a predictor of femoral IMT, we can only consider alterations in cardiovascular functions and IMT among the multitude of hyperglycemic complications in T1D. This discovery has notable implications in practice. For instance, treating hypertension alone may not be sufficient to stop femoral atherosclerosis in patients with T1D because hyperglycemia circumvents BP to act on femoral IMT.

### 4.1. Strengths and Limitations of the Study

The strengths of this study include a longitudinal follow-up of a relatively large cohort of adolescents with T1D and a comprehensive ultrasonographic evaluation of IMT at multiple arterial sites (carotid, femoral, and popliteal). This approach allowed us to compare the site-dependent magnitudes of association between IMT and HbA1c within the same individual, thereby avoiding individual-to-individual variations. The adoption of a more sophisticated statistical method known as linear mixed-effects modeling is an additional strength of our work. In contrast to other traditional methods that summarize repeated measurements, linear mixed-effects models account for correlations between repeated measurements by treating individuals as random effects. Consequently, it has greater statistical power to estimate the effects of predictor variables on the response variable. Patients with poorer diabetes control (higher HbA1c levels) tend to have elevated levels of lipids (e.g., cholesterol, triglycerides, LDL-C, sd-LDL, and cholesterol ratio), inflammation (hs-CRP), and hyperglycemia-related derangements (AGEs). This confirms our primary conclusion regarding IMT and supports similar hypotheses reported in the previous scientific literature [39, 40].

This study also has some limitations. This research was restricted by its cross-sectional design and the lack of temporal data. Therefore, we were unable to establish a causal relationship between higher HbA1c levels and greater IMT. Owing to the fact that IMT varies based on the average effects of HbA1c over time, our findings reflect a snapshot in time and do not provide a representative guarantee of the association between these factors.

## 5. Conclusion

This study supports the need for aggressive and early glucose control in patients with T1D to improve cardiovascular health. In our patient population, we also demonstrated that femoral IMT may be more sensitive than carotid or popliteal IMT for detecting subclinical atherosclerosis. Furthermore, the association between femoral IMT and the HbA1c level may not be mediated by changes in cardiovascular functions.

## Figures and Tables

**Table 1:** Baseline characteristics of adolescents with type 1 diabetes summarized according to the HbA1c levels.

Variables	Total	HbA1c (%)			*P* value^†^
		≤7	7 < •≤10	>10	
*N*	286	63	180	43	
HbA1c (%)	8.4 ± 1.9	6.3 ± 0.5	8.3 ± 0.8	11.6 ± 1.7	
HbA1c (mmol/mol)	67.8 ± 20.2	45.8 ± 4.9	66.8 ± 9.1	103.8 ± 18.4	
Age (years)	15.9 ± 4.9	14.8 ± 4.4	16.2 ± 5.1	16.2 ± 4.6	0.115
Age of onset (years)	8.5 ± 4.1	9.7 ± 4.2	8.0 ± 4.0	8.9 ± 3.8	0.145
Duration of diabetes (years)	7.5 ± 4.7	5.7 ± 4.0	8.1 ± 4.9	7.8 ± 3.7	0.008
Male (%)	42.0	44.4	43.3	32.6	0.267
BMI (kg/m^2^)	20.2 ± 3.3	19.4 ± 3.3	20.4 ± 3.3	20.6 ± 3.2	0.051
Waist (cm)	69.5 ± 9.4	67.5 ± 11.7	69.8 ± 8.4	70.8 ± 9.4	0.051
Systolic BP (mmHg)	115 ± 11	112 ± 10	115 ± 11	114 ± 11	0.255
Diastolic BP (mmHg)	68 ± 7	66 ± 7	69 ± 7	68 ± 7	0.142
Fasting glucose (mg/dL)	184 ± 79	139 ± 47	190 ± 78	229 ± 89	<0.001
TC (mg/dL)	174 ± 36	162 ± 26	176 ± 33	200 ± 44	<0.001
Triglycerides (mg/dL)	66 ± 37	56 ± 28	65 ± 35	87 ± 47	<0.001
HDL-C (mg/dL)	67 ± 14	66 ± 15	68 ± 13	67 ± 15	0.596
TC/HDL-C	2.7 ± 0.7	2.5 ± 0.6	2.7 ± 0.6	3.1 ± 1.0	<0.001
Non-HDL-C (mg/dL)	109 ± 33	96 ± 25	108 ± 30	133 ± 45	<0.001
LDL-C (mg/dL)	95 ± 32	82 ± 21	95 ± 30	116 ± 39	<0.001
sd-LDL-C (mg/dL)	19 ± 10	15 ± 5	19 ± 10	26 ± 15	<0.001
Albumin (g/dL)	4.4 ± 0.2	4.5 ± 0.2	4.4 ± 0.2	4.4 ± 0.3	0.071
Creatinine (mg/dL)	0.83 ± 0.15	0.81 ± 0.15	0.84 ± 0.15	0.78 ± 0.14	0.672
Est. CCR (mL/min)	95 ± 21	94 ± 23	94 ± 18	101 ± 24	0.138
AST (U/L)	20 ± 6	21 ± 5	20 ± 6	20 ± 6	0.122
ALT (U/L)	13 ± 6	13 ± 5	12 ± 5	15 ± 8	0.078
UA (mg/dL)	4.5 ± 1.1	4.7 ± 1.2	4.5 ± 1.1	4.3 ± 1.3	0.048
hs-CRP (mg/dL)	0.59 ± 1.21	0.23 ± 0.33	0.67 ± 1.42	0.77 ± 1.02	0.028
AGEs (AU)	1.82 ± 1.03	1.52 ± 0.66	1.78 ± 0.90	2.42 ± 1.64	<0.001

Data are presented as means ± SD for continuous variables and percentages for binary variables. ^†^*P*-value is for testing the trend with adjustment for sex and age. AGEs, advanced glycation end-products; ALT, alanine aminotransferase; AST, aspartate aminotransferase; BP, blood pressure; Est. CCR, estimated creatinine-clearance rate; HDL-C, HDL cholesterol; hs-CRP, high-sensitivity C-reactive protein; LDL-C, LDL cholesterol; sd, small-dense; TC, total cholesterol; UA, uric acid.

**Table 2:** IMTs in the carotid, femoral, and popliteal arteries based on the HbA1c levels in adolescents with type 1 diabetes.

Segments:	HbA1c (%)			*P*-value^†^
	≤7	7< and ≤10	>10	
CCA (mm)				
Right maximum	0.539 ± 0.006	0.536 ± 0.004	0.531 ± 0.008	0.478
Left maximum	0.522 ± 0.006	0.531 ± 0.004	0.526 ± 0.008	0.604
Maximum	0.530 ± 0.005	0.533 ± 0.003	0.528 ± 0.006	0.933
Right mean	0.427 ± 0.005	0.429 ± 0.003	0.425 ± 0.006	0.902
Left mean	0.416 ± 0.005	0.423 ± 0.003	0.414 ± 0.006	0.879
Mean	0.422 ± 0.004	0.426 ± 0.002	0.419 ± 0.005	0.872
ICA (mm)				
Right maximum	0.448 ± 0.008	0.455 ± 0.004	0.454 ± 0.009	0.493
Left maximum	0.434 ± 0.008	0.447 ± 0.004	0.463 ± 0.009	0.024
Maximum	0.441 ± 0.006	0.451 ± 0.003	0.458 ± 0.007	0.055
Right mean	0.377 ± 0.006	0.384 ± 0.004	0.383 ± 0.008	0.442
Left mean	0.368 ± 0.006	0.376 ± 0.004	0.375 ± 0.007	0.379
Mean	0.373 ± 0.005	0.380 ± 0.003	0.379 ± 0.006	0.310
Bulb (mm)				
Right maximum	0.508 ± 0.009	0.522 ± 0.005	0.520 ± 0.011	0.370
Left maximum	0.503 ± 0.009	0.518 ± 0.005	0.543 ± 0.011	0.009
Maximum	0.505 ± 0.007	0.520 ± 0.004	0.531 ± 0.009	0.027
Right mean	0.435 ± 0.007	0.445 ± 0.004	0.441 ± 0.009	0.544
Left mean	0.422 ± 0.008	0.436 ± 0.005	0.460 ± 0.009	0.003
Mean	0.429 ± 0.006	0.441 ± 0.004	0.451 ± 0.007	0.027
IMT maximum (mm)	0.492 ± 0.004	0.502 ± 0.003	0.506 ± 0.005	0.032
IMT mean (mm)	0.408 ± 0.004	0.416 ± 0.002	0.416 ± 0.004	0.085
CFA (mm)				
Right maximum	0.635 ± 0.022	0.664 ± 0.013	0.736 ± 0.027	0.006
Left maximum	0.618 ± 0.023	0.649 ± 0.013	0.647 ± 0.028	0.336
Maximum	0.627 ± 0.019	0.657 ± 0.011	0.701 ± 0.023	0.013
Right mean	0.550 ± 0.020	0.577 ± 0.012	0.627 ± 0.024	0.018
Left mean	0.539 ± 0.021	0.558 ± 0.012	0.559 ± 0.025	0.471
Mean	0.545 ± 0.017	0.568 ± 0.010	0.602 ± 0.021	0.032
Triple line pattern (%)	55.74	67.05	66.67	0.195
PLA (mm)				
Right maximum	0.501 ± 0.010	0.493 ± 0.006	0.534 ± 0.013	0.066
Left maximum	0.512 ± 0.010	0.520 ± 0.006	0.529 ± 0.012	0.264
Maximum	0.506 ± 0.008	0.507 ± 0.004	0.531 ± 0.009	0.050
Right mean	0.427 ± 0.009	0.419 ± 0.005	0.458 ± 0.011	0.060
Left mean	0.434 ± 0.007	0.444 ± 0.004	0.444 ± 0.009	0.291
Mean	0.430 ± 0.006	0.432 ± 0.004	0.451 ± 0.008	0.045
Triple line pattern (%)	3.28	7.51	14.29	0.042

Data are shown as means ± SD for continuous variables and as percentage for binary variables. ^†^*P* values are for testing trends with adjustment for sex and age. Abbreviations: Bulb, carotid bulb; CCA, common carotid artery; CFA, common femoral artery; ICA, internal carotid artery; PLA, popliteal artery.

**Table 3:** Multiple regression coefficients of IMT predictors by arterial sites analyzed using a linear mixed-effects model for repeated measurements.

Predictors	Sites									
	CCA		Bulb		ICA		CFA		PLA	
	Mean	Maximum	Mean	Maximum	Mean	Maximum	Mean	Maximum	Mean	Maximum
Segments^†^	–^*∗∗∗*^	–^*∗∗∗*^	—	—	–^*∗*^	—	—	—	—	—
Male vs. Female	1.33^*∗*^	0.96	1.71^*∗*^	2.52^*∗*^	2.70^*∗∗∗*^	3.01^*∗∗*^	−0.02	0.47	0.10	−0.29
Age (years)	0.58	0.88^*∗*^	1.80^*∗∗∗*^	2.18^*∗∗∗*^	1.38^*∗∗∗*^	1.40^*∗∗*^	0.29^*∗*^	0.34^*∗*^	0.27^*∗∗∗*^	0.33^*∗∗∗*^
Waist (cm)	−0.01	−0.43	1.18	1.23	1.07^*∗*^	1.04	−0.16	−0.19	0.03	0.04
BMI (kg/m^2^)	−0.08	0.64	−0.95	−1.11	−0.10	0.28	0.29	0.44	−0.13	−0.10
Systolic BP (mmHg)	0.12	0.20	0.11	0.13	−0.40	−0.46	0.01	0.03	−0.00	−0.00
HbA1c, >10% vs. ≤10%	−1.03	−0.85	0.04	0.34	−0.57	−0.12	3.95^*∗*^	4.89^*∗∗*^	0.63	0.61
LDL-C (mmol/L)	0.32	0.28	0.25	−0.01	−0.13	−0.45	−0.00	0.01	0.00	−0.00
HDL-C (mmol/L)	−0.21	−0.61^*∗*^	0.02	0.12	−0.28	−0.42	0.03	0.03	0.01	−0.00
Triglycerides (mmol/L)	−0.05	−0.44	−0.35	−0.42	0.27	0.24	0.01	0.01	0.01	0.00
Est CCr (mL/min/1.73 m^2^)	0.09	−0.02	0.03	−0.11	−0.75	−0.70	0.04	0.03	0.01	0.04
Hemoglobin (g/dL)	−0.23	0.07	0.12	−0.13	0.01	0.05	−0.51	−0.75	0.03	0.16
hs_CRP (mg/dL)	−0.04	0.13	0.39	0.88^*∗*^	0.25	0.13	0.06	−0.06	−0.03	0.07
AGEs (unit/mL)	0.10	0.68	0.49	0.70	0.21	0.10	−0.04	−0.32	−0.15	0.06
Urine ACR (mg/g)	0.50^*∗*^	0.65^*∗*^	−0.23	−0.44	0.02	0.02	−0.00	−0.00	−0.00	−0.00

Values are presented as the original values × 100. ^*∗*^*P* < 0.05, ^*∗∗*^*P* < 0.01, ^*∗∗∗*^*P* < 0.001. ^†^A “segments” variable is introduced into the mixed-effects model to account for arterial segments (bilateral CCAs, Bulbs, and ICAs for the carotid site; bilateral CFAs for the femoral site; and bilateral PLAs for the popliteal site). Interaction terms were retained in the final model if significant. ACR, albumin/creatinine ratio; AGEs, advanced glycation end-products; BP, blood pressure; Est CCr, estimated creatinine clearance rate; hs-CRP, high-sensitivity C-reactive protein.

**Table 4:** Cardiac and vascular parameters measured by DynaPulse in adolescents with type 1 diabetes by the HbA1c levels.

Characteristics	HbA1c (%)			*P*-value for trend
	≤7	7< and ≤10	>10	
	*n* = 63	*n* = 180	*n* = 43	
Systolic BP (mmHg)	96 ± 18	104 ± 11	103 ± 9	0.0001
Diastolic BP (mmHg)	59 ± 12	63 ± 8	63 ± 7	0.0006
Central systolic BP (mmHg)	112 ± 10	115 ± 11	115 ± 11	0.0047
Central diastolic BP (mmHg)	66 ± 7	69 ± 7	68 ± 7	0.5575
Mean artery pressure (mmHg)	73 ± 9	77 ± 8	76 ± 7	0.0037
Pulse pressure (mmHg)	50 ± 10	51 ± 9	51 ± 9	0.1568
Heart rate (beats/min)	77 ± 14	79 ± 12	85 ± 8	0.0403
LV ejection time (s)	0.26 ± 0.07	0.27 ± 0.05	0.26 ± 0.03	0.0005
LV d*p*/d*t* max (mmHg/s)	1159 ± 208	1206 ± 190	1213 ± 197	0.0006
LV contractility (1/s)	16.94 ± 3.44	17.59 ± 2.05	18.10 ± 1.54	0.1238
Cardiac output (L/min)	4.44 ± 1.34	4.84 ± 1.06	5.30 ± 0.95	0.0548
Cardiac index (L/min/m^2^)	3.10 ± 0.84	3.32 ± 0.64	3.61 ± 0.49	0.3617
Stroke volume (mL)	58 ± 16	61 ± 11	62 (11)	0.0459
Stroke volume index (mL/m^2^)	40 ± 8	42 ± 5	42 ± 4	0.9213
Systemic vascular compliance (mL/mmHg)	1.18 ± 0.30	1.23 ± 0.24	1.25 ± 0.20	0.6314
Systemic vascular resistance (dynes/s/cm^5^)	1230 ± 319	1275 ± 226	1177 ± 167	0.0947
Brachial artery compliance (mL/mmHg)	0.05 ± 0.02	0.06 ± 0.02	0.05 ± 0.02	0.0172
Brachial artery distensibility (%/mmHg)	7.02 ± 1.95	6.80 ± 1.43	6.90 ± 1.47	0.2464
Brachial artery resistance (kdynes/s/cm^5^)	284 ± 106	275 ± 120	263 ± 122	0.6632

Data are presented as means ± SDs. LV, left ventricle.

## Data Availability

The datasets used to support the findings of this study are available upon request from the corresponding author.
